# ﻿*Chimonobambusasangzhiensis* (Poaceae, Bambusoideae), a new combination supported by morphological and molecular evidence

**DOI:** 10.3897/phytokeys.195.83004

**Published:** 2022-05-09

**Authors:** Zheng-Yang Niu, Zhuo-Yu Cai, Chun-Lin Liao, Nian-He Xia

**Affiliations:** 1 Key Laboratory of Plant Resources Conservation and Sustainable Utilization & Guangdong Provincial Key Laboratory of Digital Botanical Garden, South China Botanical Garden, Chinese Academy of Sciences, 510650, Guangzhou, China South China Botanical Garden, Chinese Academy of Sciences Guangzhou China; 2 University of Chinese Academy of Sciences, 100049, Beijing, China University of Chinese Academy of Sciences Beijing China; 3 National Nature Reserve of Badagongshan, Sangzhi, Hunan, China National Nature Reserve of Badagongshan Sangzhi China

**Keywords:** bamboo, morphology, new combination, phylogeny

## Abstract

This study elucidates the taxonomic position of *Indosasasangzhiensis* in considering whether it belongs to *Indosasa* or *Chimonobambusa*. Based on morphological and molecular phylogenetic evidence, our results explicitly indicated that *I.sangzhiensis* should be a member of *Chimonobambusa*, rather than *Indosasa*, and is a distinct species closely related to *C.communis*, *C.opienensis* and *C.puberula*. Thus, the new combination *Chimonobambusasangzhiensis* (B.M.Yang) N.H.Xia & Z.Y.Niu is made. A detailed description as well as two color plates of this species are also provided.

## ﻿Introduction

*Chimonobambusa*Makino (1914), belonging to the subtribe Arundinariinae of the tribe Arundinarieae (Poaceae, Bambusoideae) (Zhang et al 2020), is characterized by leptomorph rhizomes, diffuse culms, basal internodes that are 4-angled and frequently with a ring of root thorns, internodes with 2 longitudinal ridges and 3 grooves just above branching points at the node, three branches at each mid-culm node, small, triangular or subulate culm leaf blades (usually less than 1 cm long), pseudospikelets, and 3 stamens in each floret (Makino 1914; Xue and Zhang 1996; Li and Stapleton 2006). It contains about 42 species mainly distributed in China, Japan, Myanmar and Vietnam (Vorontsova et al. 2016). There are 38 species known, mainly distributed in Central, South and Southeast China (Li and Stapleton 2006; Vorontsova et al. 2016). Recent phylogenetic studies that focused on Arundinariinae have suggested that *Chimonobambusa* should be defined in a broad sense, including *Qiongzhuea* Hsueh & T.P.Yi (Hsueh and Yi 1980; Xue and Yi 1996), and thus it can be accepted as a monophyletic group, except for *C.sichuanensis* (T.P.Yi) T.H.Wen (Li and Stapleton 2006; Peng et al. 2008; Zhang et al. 2012; Guo et al. 2021).

*Indosasasangzhiensis* B.M.Yang was described based on a collection (Vegetation Survey Group 00549) from Badagongshan, Sangzhi Xian, Hunan Province, China. In the protologue, Yang (1989) stated that this species resembled *I.glabrata* C.D.Chu & C.S.Chao (Chao and Chu 1983), but can be distinguished by its densely pubescent culms, solid distal internodes but hollow basal ones, few foliage leaf oral setae, as well as small and adaxially pubescent foliage leaf blades. However, after examination of the type specimen and protologue of *I.sangzhiensis*, we found that this species is characterized by small, erect and triangular culm leaf blades, which falls within the circumscription of *Chimonobambusa* rather than *Indosasa*.

During fieldwork at the type locality of *I.sangzhiensis* (Badagongshan, Sangzhi), we collected a bamboo with leptomorph rhizomes, diffuse culms and three branches at mid-culm nodes. After comparison of the specimens we collected and possibly related species, we found that it matches the type of *I.sangzhiensis* in both having culm internodes with densely white pubescence, solid distal internodes but hollow basal ones, ovate culm buds, small, erect and triangular culm leaf blades, glabrous culm leaf sheaths, 2–3 leaves per ultimate branches, developed foliage leaf oral setae and leaf blades with length of 9–19 cm and width of 1.2–2 cm. Therefore, we concluded that the specimens we collected are *I.sangzhiensis*. In addition, we found that this species is characterized by internodes with two longitudinal ridges and three grooves above the branching point, slightly 4-angled basal internodes, flat or only slightly prominent nodes, small, erect and narrowly triangular or subulate culm leaf blades, which is not typical of *Indosasa* species as far as we know, but conforms well with the known morphology of *Chimonobambusa*. Therefore, we conclude that *I.sangzhiensis* should be a member of *Chimonobambusa*, rather than *Indosasa*. After checking records of *Chimonobambusa* species from the Flora of China, we found that *I.sangzhiensis* is closely related to three species, viz., *Chimonobambusacommunis* (Hsueh & T.P.Yi) T.H.Wen & Ohrnb., *C.opienensis* (Keng f.) T.H.Wen & Ohrnb. and *C.puberula* (Keng f.) T.H.Wen & Ohrnb (Hsueh and Yi 1980, 1983; Hsueh et al. 1996). In order to elucidate the relationship between *I.sangzhiensis* and related species, a detailed comparison of vegetative morphological characters and phylogenetic analyses were conducted in this study.

## ﻿Materials and methods

### ﻿Morphological observation

The main morphological characters of *I.sangzhiensis* and three species of *Chimonobambusa*, viz., *C.communis*, *C.opienensis* and *C.puberula*, were compared based on protologues and descriptions from floras. Some detailed characters, such as indumentum of the culm leaf ligule, were observed with a stereomicroscope (Mshot MZ101). Measurements were taken using a ruler or micrometer.

### ﻿Taxon sampling for phylogenetic analyses

Two different molecular regions including complete chloroplast genome (cpDNA) and nuclear ribosomal DNA (nrDNA) were utilized to reconstruct the phylogenetic relationships of *I.sangzhiensis*. A total of 23 species representing 7 genera from Arundinarieae were utilized to reconstruct the plastid tree, for which *Bambusaemeiensis* L.C.Chia & H.L.Fung and *B.sinospinosa* McClure were set as the outgroup taxa. For nrDNA, 16 species representing 5 genera from Arundinarieae were utilized to reconstruct the nrDNA tree, for which *B.sinospinosa* and *B.multiplex* (Lour.) Raeusch. ex Schult.f. were set as the outgroup taxa. The generic type of *Indosasa* McClure, *I.crassiflora* McClure, was added here to clarify the systematic position of *I.sangzhiensis*. All voucher information and accession number of cp genomes are listed in Table 1. Our sample information for nrDNA and its sequence matrix can be found in the supplementary material (Suppl. material 1: Table S1).

**Table 1.** Voucher information of 22 complete chloroplast genomes used in this study.

Taxon	Voucher information	GenBank accession
**Ingroup**		
*Acidosasapurpurea* (Hsueh & T.P.Yi) Keng f.	Zhang08023 (KUN)	HQ337793
*Chimonobambusaangustifolia* C.D.Chu & C.S.Chao	Wu20210053(YAFG)	OK040768
*C.hejiangensis* C.D.Chu & C.S.Chao	GACP (NMGU)	MT884004
*C.hirtinoda* C.S.Chao & K.M.Lan	Not provided by the author	MT576658
*C.purpurea* Hsueh & T.P.Yi	LW20200602-01 (CAAF)	MW030500
*C.tumidissinoda* Ohrnb.	MPF10083 (KUN)	MF066244
*C.utilis* (Keng) Keng f.	Not provided by the author	OK040769
*Indocalamussinicus* (Hance) Nakai	ZMY037 (KUN)	MF066250
*I.tongchuensis* K.F.Huang & Z.L.Dai	Not provided by the author	MW279198
*Ravenochloawilsonii* (Rendle) D.Z.Li & Y.X.Zhang	MPF10146 (KUN)	JX513421
*Indosasacrassiflora* McClure	BH58 (IBSC)	OK558536
*I.gigantea* (T.H.Wen) T.H.Wen	HNJ36052 (JXAU)	MN917206
*I.sangzhiensis* B.M.Yang	NZY109 (IBSC)	OM867788
*I.shibataeoides* McClure	MPF10028 (KUN)	MF066251
*I.sinica* C.D.Chu & C.S.Chao	MPF10034 (KUN)	MH394381
*Oligostachyumshiuyingianum* (L.C.Chia & But) G.H.Ye & Z.P.Wang	DZL09122 (KUN)	JX513423
*O.sulcatum* Z.P.Wang & G.H.Ye	Not provided by the author	MW190089
*Pleioblastusamarus* (Keng) Keng f.	Zhang Yu-QuC373 (SANU)	MH988736
*P.maculatus* (McClure) C.D.Chu & C.S.Chao	MPF10161 (KUN)	JX513424
*P.triangulata* (Hsueh & T.P.Yi) N.H.Xia, Y.H.Tong & Z.Y.Niu	NZY040 (IBSC)	OK323193
**Outgroup**		
*Bambusaemeiensis* L.C.Chia & H.L.Fung	Zhang08019 (KUN)	HQ337797
*B.sinospinosa* McClure	Li043 (KUN)	MK679807

### ﻿DNA extraction, sequencing, assembly and annotation

Total genomic DNA was isolated from silica-dried leaves following manufacturer specifications TIANGEN Genomic DNA Extraction Kit (TIANGEN, Beijing, China). DNA samples of concentration up to standard (≥1 μg) were randomly sheared into fragments using Covaris M220 (Covaris, Woburn, MA). Insert size of 350 bp fragments were enriched by PCR, and the paired-end (2 × 150 bp) libraries were constructed on NovaSeq 6000 platform. As a result, about 20 G genome skimming data were generated.

To improve assembly accuracy and efficiency, Trimmomatic v 0.39 were utilized to filter out unpaired and low-depth reads from clean data using default parameters (Bolger et al. 2014). The whole cpDNA and nrDNA were assembled using the software GetOrganelle v 1.7.4 pipeline (Jin et al. 2018), with five k-mer sets of 45, 65, 85, 105, 125 bp. The filtered reads were transferred to Bandage (Wick et al. 2015) for plastid and ribosomal DNA scaffolds connection. Two opposite plastid scaffolds exported from Bandage were aligned with the reference *I.shibataeoides* (MF066251), and the one matching the reference was annotated using the PGA software (Qu et al. 2019) based on the annotation of *I.shibataeoides*. The sequences were finally checked manually in Geneious 9.1.4 (Kearse et al. 2012). The assembled nrDNA sequences were annotated directly using Geneious. Illustration of the newly sequenced plastome of *I.sangzhiensis* was drawn by OGDRAW with default settings (Greiner et al. 2019).

### ﻿Phylogenetic analysis

To determine the systematic position of *I.sangzhiensis*, maximum likelihood (ML) and Bayesian inference (BI) analyses were conducted. A total of 22 complete cp and 16 nrDNA genomes were aligned with MAFFT v 7.450 (Katoh and Standley 2013). Maximum likelihood (ML) analysis was generated by RAxML v 8.0.0 (Stamatakis 2014). Rapid bootstrap analysis and GTRGAMMAI were set as the best-fit algorithm and model. The number 12345 was specified as the random seed of parsimony tree inference with 1000 replicates performed. Bayesian inference (BI) was conducted using MrBayes v 3.2.6 (Ronquist et al. 2012). The model of SYM+G was defined by MrModeltest v 2.4 (Nylander 2004). The rates of variations across sites were trimmed as gamma. At least 6,000,000 generations were run to ensure average standard deviation of split frequencies (ASDFs) < 0.01 with sampling frequency set as 100 generations. After rejecting the first 25% burn-in samples, the optimized topology was printed.

## ﻿Results

### ﻿Basic features of plastome and nrDNA

**Figure 1.**
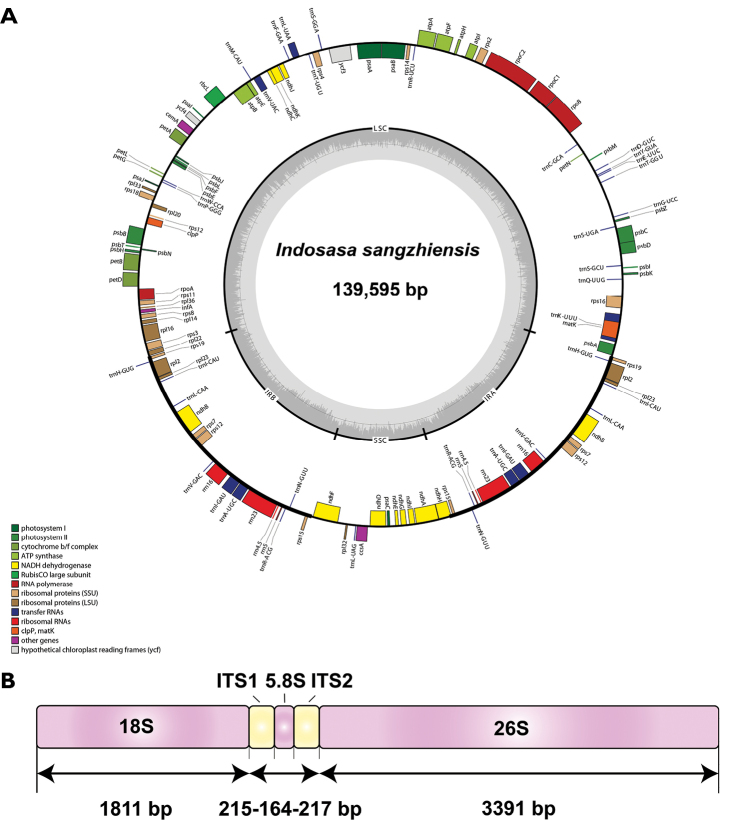
The plastome map **A** and nrDNA structure **B** for *I.sangzhiensis*.

The plastid genome of *I.sangzhiensis* exhibited a typical quadripartite structure. Its genome size is 139,595 bp including a large single copy region (LSC) of 83,190 bp, a small single copy region (SSC) of 12,811 bp and a pair of inverted repeat regions (IRs) of 21,797 bp (Fig. 1A). The overall GC content is 38.9%. The whole plastid genome contains 108 unique genes, including 76 protein-coding genes, 28 transfer RNAs and 4 ribosomal RNAs. Among them, 43 genes are associated with photosynthesis and 59 genes are relevant to gene expression.

For the tandemly repeated nrDNA, our *de novo* assembly obtained 5,799 bp sequences comprising 18S (1,811 bp), 5.8S (164 bp), and 26S (3,391 bp) ribosomal RNA gene along with two internal transcribed spacer I (ITS1) (215 bp) and ITS2 (217 bp) in the middle (Fig. 1B).

### ﻿Morphological comparison

**Figure 2.**
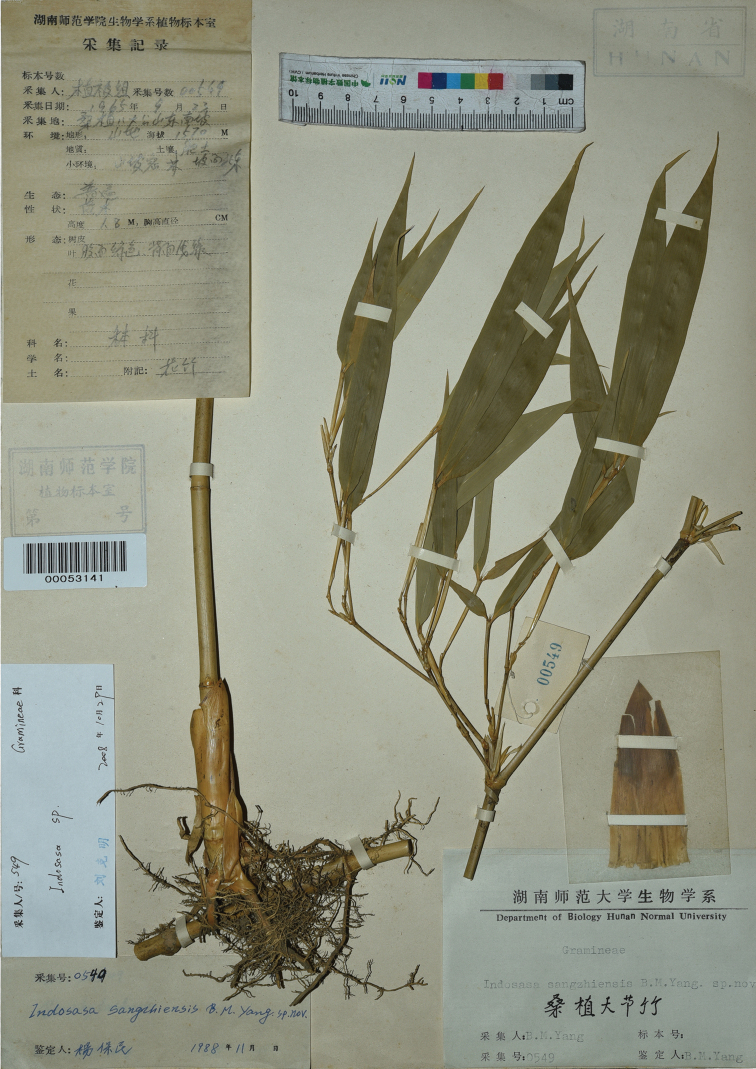
The holotype of *I.sangzhiensis* B.M.Yang (HNNU).

**Figure 3.**
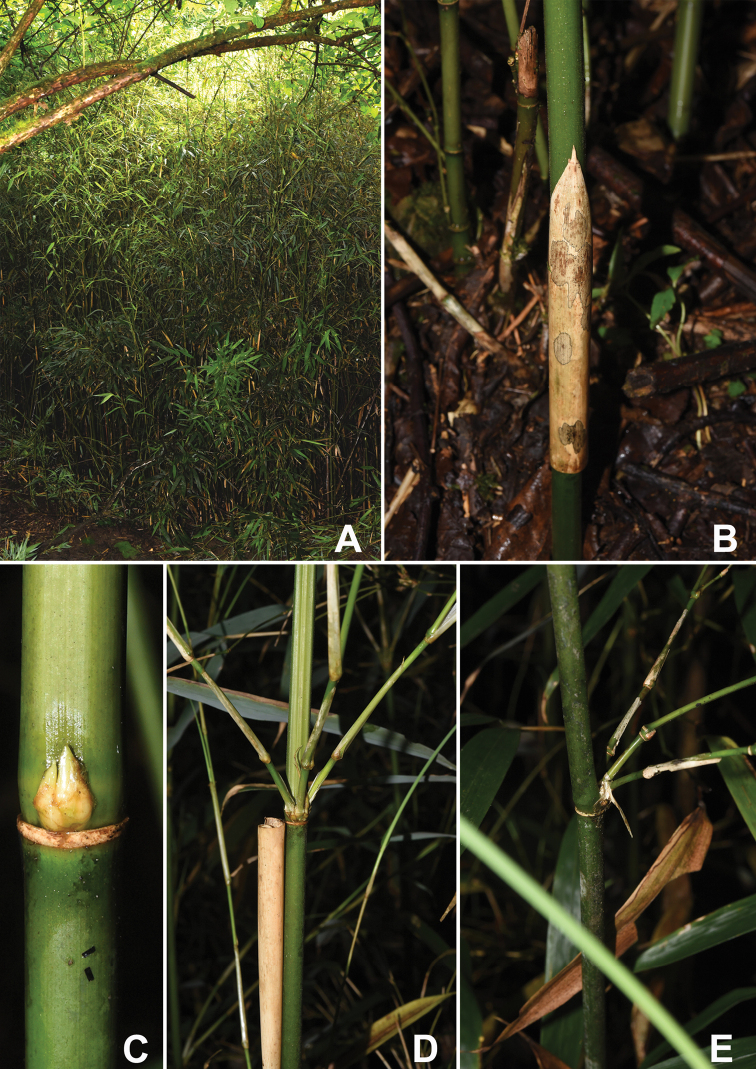
Morphological characters of *I.sangzhiensis***A** habit **B** culm leaf sheath on internodes covered with dense white pubescence **C** culm bud, node and sheath scar **D** two longitudinal ridges and three grooves above branches **D, E** branches at mid-culm nodes, young **D** and old **E** culm. All photos by Z.Y.Niu.

*Indosasasangzhiensis* resembles *C.communis*, *C.opienensis* and *C.puberula* in having slightly 4-angled basal culm internodes, flat or slightly prominent culm nodes without root thorns, deciduous culm leaf sheaths, very small, erect and narrowly triangular or subulate culm leaf blades and well-developed foliage leaf oral setae; but can be easily distinguished from the latter three species by the morphological characters shown in Table 2. Specifically, it can be distinguished from *C.communis* in having culm internodes with dense white pubescence (Fig. 2B–E) (vs. glabrous internodes), culm leaf ligule margins with dense cilia (Fig. 3C) (vs. glabrous margin) and culm leaf blades broadly extending to join the sheath apex (Fig. 3B) (vs. slightly narrowed at the junction with the sheath apex). It differs from *C.opienensis* in having culm internodes with dense white pubescence (Fig. 2B–E) (vs. glabrous), 3 branches per mid-culm nodes (Fig. 2D, E) (vs. 2–3 branches per mid-culm node), culm leaf sheaths of glabrous adaxial surface (Fig. 3A) (vs. with sparse brown setose), culm leaf ligule margins with dense cilia (Fig. 3C) (vs. glabrous) and 1–3 foliage leaves per ultimate branch (Fig. 3D) (vs. 1(–2) foliage leaves). It differs from *C.puberula* in having glabrous rhizome internodes (Fig. 3E) with densely brown-setose parts below the nodes and glabrous rhizome internodes sheath scars (Fig. 2C) (vs. with densely brown setose); culm leaf sheaths with glabrous abaxial surface (Fig. 3A) (vs. with densely brown setose surface); culm leaf ligule margins with dense cilia (Fig. 3C) (vs. glabrous margin) and new shoots produced during April to June (vs. October).

**Table 2.** Morphological comparison of *Indosasa. Sangzhiensis* and three related species.

Morphology	*I.sangzhiensis*	*C.communis*	*C.opienensis*	*C.puberula*
Rhizome				
Surface of infranodes	Glabrous	Glabrous	Glabrous	Densely brown setose
Culm				
Height (m)	1–3	3–7	2–7	4–5
Diameter (mm)	0.5–1.5	1–3	1–5.5	1.5–2.5
Surface of internodes	Densely pubescent	Glabrous	Glabrous	Densely pubescent
Sheath scar	Glabrous	Glabrous	Glabrous	Densely brown setose
Branches				
Number per mid-culm node	3	3	2 or 3	3
Culm leaf				
Abaxial surface of sheaths	Glabrous	Glabrous	Sparsely brown setose	Densely brown setose
Apex of ligule	Densely ciliate	Glabrous	Glabrous	Glabrous
Base of blade	Extending outward to join sheath apex	Slightly narrowed to join sheath apex	Extending outward to join sheath apex	Extending outward to join sheath apex
Foliage leaf				
Number per ultimate branch	1–3	1–3	1(–2)	2–4
Shoots				
Phenology	April to June	May	April to May	October

**Figure 4.**
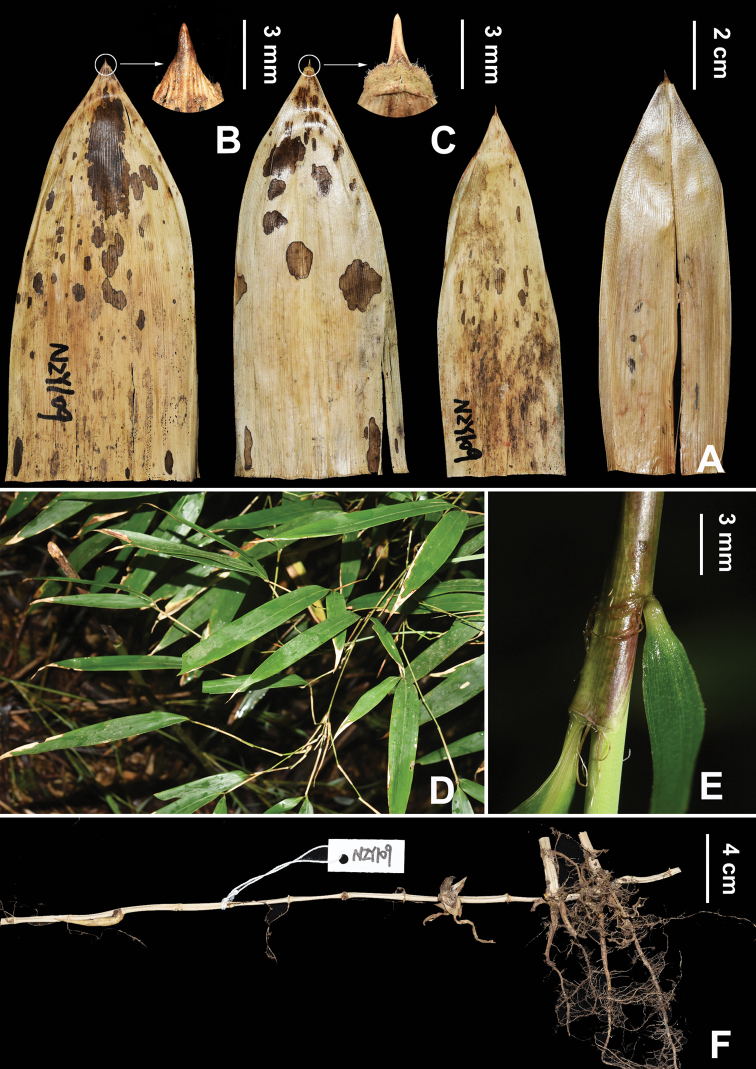
Morphological characters of *I.sangzhiensis***A** culm leaf, detached **B** culm leaf blade **C** culm leaf ligule **D** ultimate leafy branches **E** foliage leaf oral setae **F** rhizome with partial young culms. All photos by Z.Y.Niu.

### ﻿Phylogenetic analysis

**Figure 5.**
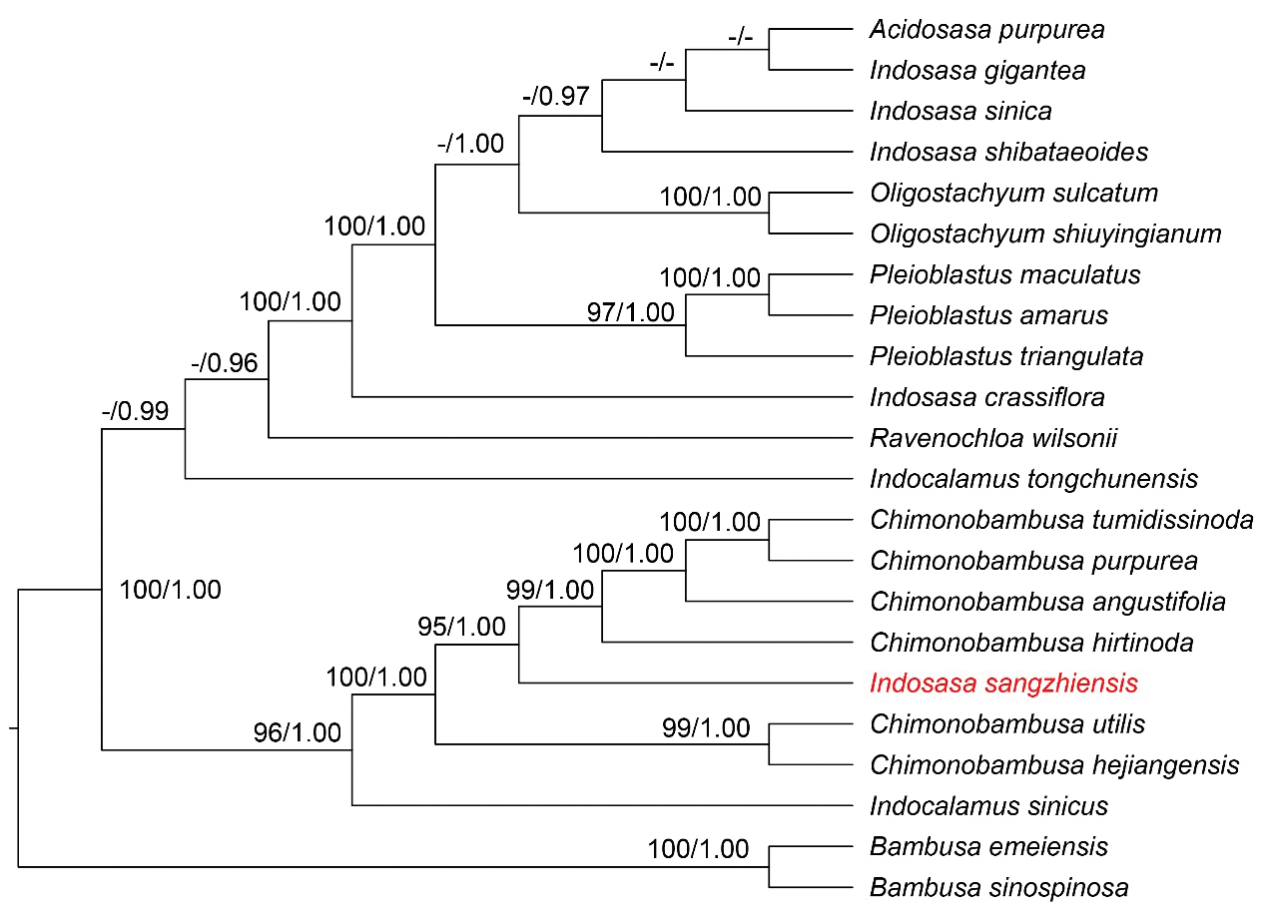
Phylogenetic tree reconstruction for *I.sangzhiensis* based on plastid genome dataset with maximum likelihood and Bayesian analyses. Only bootstrap values (BS) ≥ 70% and posterior probabilities (PP) ≥ 0.95 are indicated at each node.

**Figure 6.**
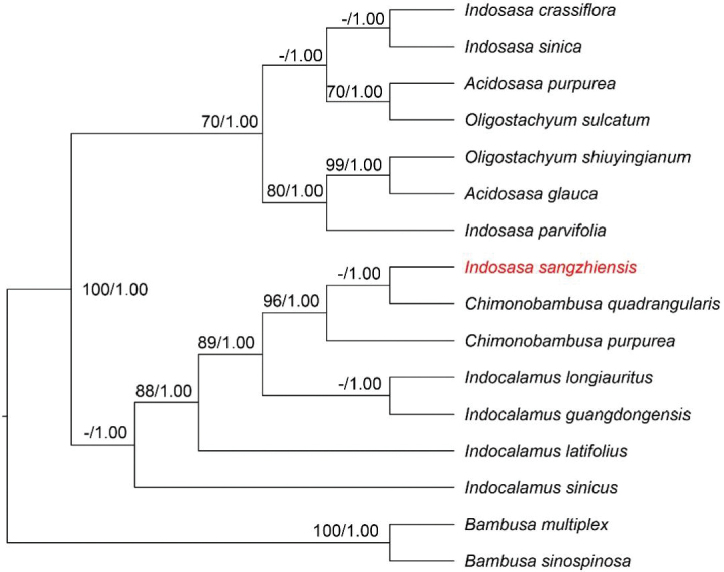
Phylogenetic tree reconstruction for *I.sangzhiensis* based on nrDNA dataset by using maximum likelihood analysis and Bayesian analysis. Only bootstrap values (BS) ≥ 70% and posterior probabilities (PP) ≥ 0.95 are indicated at each node.

The topologies based on ML and BI methods did not indicate any conflict between the cpDNA and nrDNA phylogenetic analyses, thus only ML cladograms are shown here (Figs 5, 6), with BP and PP values noted at each node. The plastid and nrDNA phylogenetic trees both strongly supported the case that *I.sangzhiensis* is distantly related to *I.crassiflora* (the type of *Indosasa*) (BS = 100% & PP = 1.00), but forms a sister clade with members of *Chimonobambusa* (BS = 95% & PP = 1.00) (Fig. 5). Our results also indicate that all samples of *Chimonobambusa* formed a monophyletic clade while *Indosasa* was found to be polyphyletic, as the samples of *Indosasa* and those of two other genera, viz. *Acidosasa* and *Oligostachym*, were intermixed within a clade.

## ﻿Discussion

*Indosasa* was published by McClure (1940) based on a collection of flowering material in Vietnam. It is conventionally defined by having leptomorph rhizomes, diffuse culms, three branches per mid-culm node, prominent nodes, pseudospikelets and six stamens per floret (McClure 1940; Zhu and Stapleton 2006). Until now, it was thought that there were 18 species of *Indosasa* mainly distributed in South, Southwest and East China, Vietnam and Laos (Zhu and Stapleton 2006; Vorontsova et al. 2016; Niu et al. 2021). However, recent molecular evidence based on chloroplast genome and ddRAD data indicated that *Indosasa* is a highly polyphyletic group placed in the subtribe Arundinariinae of the tribe Arundinarieae (Ma et al. 2017; Zhang et al. 2020), and the phylogenetic relationships among *Indosasa* and several closely related genera with similar vegetative characters, such as *Acidosasa*, *Oligostachyum*, *Sinobambusa*, etc., have not been resolved (Guo et al. 2021; Niu et al. 2021). However, a broad concept was proposed for *Chimobambusa* based on recent molecular evidence (Li and Stapleton 2006; Peng et al. 2008; Zhang et al. 2012; Guo et al. 2021), and hence this genus has been assumed to be monophyletic so far. Although *Chimonobambusa* resembles *Indosasa* in having leptomorph rhizomes, diffuse culms, three branches at each mid-culm node and pseudospikelets, it can still be distinguished from *Indosasa* in having slightly 4-angled basal internodes, internodes with two longitudinal ridges and three grooves above the branching point, basal nodes often with a ring of root thorns, small, triangular or subulate culm leaf blades (usually less than 1 cm long) and each floret with 3 stamens. In this study, based on the results of morphological and phylogenetic analyses, we consider that *I.sangzhiensis* should be a distinct species of *Chimonobambusa*, rather than *Indosasa*, and thus, a new combination in *Chimonobambusa* is proposed.

### ﻿Taxonomic treatment

#### ﻿ Chimonobambusa sangzhiensis

Taxon classificationPlantaePoalesPoaceae

(B.M.Yang) N.H.Xia & Z.Y.Niu comb. nov.

FB8A6704-90A8-519F-95FC-2E17BBF54590

urn:lsid:ipni.org:names:77297484-1

Figs 2 , 3 , 4

##### Basionym.

*Indosasasangzhiensis*B.M. Yang (1989: 333); Yang (1993: 90); Yi et al. (2021: 244).

##### Type.

China. Hunan: Sangzhi Xian, Badagongshan, el. 1570 m, 23 September 1965, Vegetation Survey Group 00549 (holotype: HNNU!).

##### Note.

After examining the type specimen and other specimens collected from the type locality, we are able to provide a revised description of the morphology of this species below.

##### Description.

Small sized bamboo. Rhizomes leptomorph; internodes cylindrical, 4–angled when dry, 1.5–4 cm long, 5–8 mm in diameter, hollow, glabrous, walls ca. 1 mm thick; nodes flat, 1–3 roots at each node, glabrous; rhizome buds broad-ovate to subrounded, glabrous. Culms diffuse, erect, 1–3 m tall and 0.5–1.5 cm in diameter; internodes terete or base slightly 4–angled, with two longitudinal ridges and three grooves above branching points, 15–20(–24) cm long, densely white puberulent, especially below nodes, mid-culm internodes hollow, walls 1.5–3 mm thick, upper and basal internodes nearly solid; basal nodes without root thorns; supranodal ridges flat or slightly prominent at unbranched nodes, sheath scars prominent, corky, glabrous, intranodal regions 2–5 mm long, glabrous. Primary buds solitary, ovate, yellowish green, 5–8 × 3–5 mm, glabrous. Mid-culm branch complement with 3 branches, erect, subequal, inclined at an angle of 15°–45° with the culm, internodes 1.5–10 cm long, glabrous; supranodal ridges prominent; branch leaf sheaths thinly leathery, shorter than internodes, abaxially glabrous. Culm leaf sheaths long triangular, caducous, thinly leathery, 10–15 × 3.5–6 cm, 1/2–3/4 as long as internodes, pale brown, abaxially glabrous, longitudinal ribs conspicuous, upper parts of margins sparsely ciliate, deciduous when old; auricles absent; oral setae several, curly, scabrid, pale brown, 2–5 mm long, deciduous when old; ligules arcuate to truncate, 0.5–1.5 mm long, entire, densely ciliate; culm leaf blades erect, narrowly triangular or subulate, 4–11 × 1.5–4 mm, middle and upper parts involute, base extending broadly outward to join sheath apex, both sides glabrous, longitudinal ribs conspicuous. Foliage leaves 1–3 per ultimate branch; foliage leaf sheaths 3–4 cm long, initially purplish red, later becoming green, abaxially pubescent, glabrescent, margins ciliate, longitudinal ribs conspicuous; auricles absent; oral setae 3–5, curly, scabrid, 2–5 mm long; ligule truncate, ca. 1 mm long, entire or sparsely ciliate; pseudo-petioles 2–5 mm long, sparsely ciliate; foliage leaf blades lanceolate, papery, 9–19 × 1.2–2 cm, base widely cuneate, apex acute, abaxially glabrous, adaxially sparsely pubescent, glabrescent, margins serrulate, longitudinal secondary veins 3–5 pairs, transverse veins conspicuous. Inflorescence unknown.

##### Phenology.

New shoots produced during April to June.

##### Vernacular names.

Lěng Zhú (Chinese pronunciation), 冷竹 (Chinese name).

##### Distribution and habitat.

The species has only been found at its type locality so far and is rather common on mountains between elevations of 1600 m to 1900 m. It prefers a cold and moist environment and often grows under forest cover.

##### Additional specimen examined.

The same locality as the type, along the forest path, 29°40'43"N, 109°44'60"E, 1610 m, 29 June 2021, *Z.Y. Niu* NZY109 (IBSC).

## Supplementary Material

XML Treatment for Chimonobambusa sangzhiensis

## Supplementary materials

Supplementary material 1 Table S1 Data type Sample information. Explanation note Number and voucher of 16 nrDNAs. https://binary.pensoft.net/file/681740This dataset is made available under the Open Database License (http://opendatacommons.org/licenses/odbl/1.0/). The Open Database License (ODbL) is a license agreement intended to allow users to freely share, modify, and use this Dataset while maintaining this same freedom for others, provided that the original source and author(s) are credited.Zheng-Yang Niu, Nian-He Xia

Supplementary material 2 *Chimonobambusasangzhiensis* nrDNA Data type nrDNA sequences (fasta. file). Explanation note nrDNA sequences of 16 species sampled in this study. https://binary.pensoft.net/file/681741This dataset is made available under the Open Database License (http://opendatacommons.org/licenses/odbl/1.0/). The Open Database License (ODbL) is a license agreement intended to allow users to freely share, modify, and use this Dataset while maintaining this same freedom for others, provided that the original source and author(s) are credited.Zheng-Yang Niu, Nian-He Xia
